# A View on Genomic Medicine Activities in Africa: Implications for Policy

**DOI:** 10.3389/fgene.2022.769919

**Published:** 2022-04-27

**Authors:** C. Victor Jongeneel, Maritha J. Kotze, Archana Bhaw-Luximon, Faisal M. Fadlelmola, Yasmina J. Fakim, Yosr Hamdi, Samar Kamal Kassim, Judit Kumuthini, Victoria Nembaware, Fouzia Radouani, Nicki Tiffin, Nicola Mulder

**Affiliations:** ^1^ Independent Consultant, Rolle, Switzerland; ^2^ Division of Chemical Pathology, Department of Pathology, Faculty of Medicine and Health Sciences, Stellenbosch University and National Health Laboratory Service, Tygerberg Hospital, Cape Town, South Africa; ^3^ Center for Biomedical and Biomaterials Research, University of Mauritius, Reduit, Mauritius; ^4^ Centre for Bioinformatics and Systems Biology, Faculty of Science, University of Khartoum, Khartoum, Sudan; ^5^ Biotechnology Unit, Faculty of Agriculture, University of Mauritius, Reduit, Mauritius; ^6^ Laboratory of Biomedical Genomics and Oncogenetics, Institut Pasteur de Tunis, Universite’ Tunis El Manar, Tunis, Tunisia; ^7^ Laboratory of Human and Experimental Pathology, Institut Pasteur de Tunis, Tunis, Tunisia; ^8^ Medical Biochemistry and Molecular Biology Department, Faculty of Medicine, Ain Shams University and MASRI Research Institute, Ain Shams University, Cairo, Egypt; ^9^ South African National Bioinformatics Institute (SANBI), Life Sciences Building, University of Western Cape (UWC), Cape Town, South Africa; ^10^ Division of Human Genetics, Department of Pathology, Faculty of Health Sciences, University of Cape Town, Cape Town, South Africa; ^11^ Chlamydiae and Mycoplasmas Laboratory, Research Department, Institut Pasteur du Maroc, Casablanca, Morocco; ^12^ Computational Biology Division, Department of Integrative Biomedical Sciences, IDM, University of Cape Town Faculty of Health Sciences, Cape Town, South Africa; ^13^ Wellcome CIDRI-Africa, University of Cape Town Faculty of Health Sciences, Cape Town, South Africa

**Keywords:** Africa, genomic medicine, infrastructure, capacity development, readiness checklist, precision medicine stakeholders, translational research, Pathology-supported genomics

## Abstract

Genomics policy development involves assessing a wide range of issues extending from specimen collection and data sharing to whether and how to utilize advanced technologies in clinical practice and public health initiatives. A survey was conducted among African scientists and stakeholders with an interest in genomic medicine, seeking to evaluate: 1) Their knowledge and understanding of the field. 2) The institutional environment and infrastructure available to them. 3) The state and awareness of the field in their country. 4) Their perception of potential barriers to implementation of precision medicine. We discuss how the information gathered in the survey could instruct the policies of African institutions seeking to implement precision, and more specifically, genomic medicine approaches in their health care systems in the following areas: 1) Prioritization of infrastructures. 2) Need for translational research. 3) Information dissemination to potential users. 4) Training programs for specialized personnel. 5) Engaging political stakeholders and the public. A checklist with key requirements to assess readiness for implementation of genomic medicine programs is provided to guide the process from scientific discovery to clinical application.

## Introduction

In February 2021, on commission from the African Academy of Sciences, a group of scientists including the authors of this Policy Brief published a White Paper entitled “A Framework for the Implementation of Genomic Medicine for Public Health in Africa” (Policy Paper: A Framework for the Implementation of Genomic Medicine for Public Health in Africa | The AAS). This framework outlines the challenges faced by African stakeholders aiming to implement genomic medicine in their health care systems, and makes specific recommendations to address these challenges in the areas of infrastructure, the selection of participants, the collection of clinical and demographic data, the actionability of linkages between genotypes and phenotypes, ethical legal and social implication (ELSI) issues and data governance, education and training, translation of research to clinical practice, and stakeholder engagement. As a supplement to the preparation of the framework document, a Task Force was formed to conduct a survey during 2019 and 2020 that would provide a glance of the state of genomic medicine programs in Africa.

Precision medicine for improved public health relies on implementation of prevention and treatment strategies informed by the combination of genetic, environmental, and social factors, which could be targeted to individual genomes and/or the population genetic background (Molster et al., 2018). Based on the responses of representative stakeholders to 25 questions, the questionnaire sought to evaluate the knowledge of the respondents, their institutional environment, the penetration of genomic medicine approaches in their home institution, the awareness of the field in their country, and their opinion on the obstacles faced during implementation initiatives. The full questionnaire is available at http://j.mp/37YDNuw and included in the Supplementary Material. Ethics approval for this research was obtained from the Human Research Ethics Committee of the University of Cape Town in South Africa. Although a request to take the survey was sent to many African stakeholders from academic institutions, government and private industry, only 78 responses were received, of which 55 were complete enough to be included in our analysis. While this is not sufficient to draw any truly robust conclusions, we believe that enough information was provided by the respondents to detect useful trends and, along with the aforementioned White Paper, to develop some recommendations to inform policy.

Here, the policy-related implications of responses that were received from twelve African countries are discussed, with the aim to provide a checklist with key requirements to assess readiness for implementation of genomic medicine programs. A more quantitative analysis of the survey results, as much as was possible with the limited responses, is included as Supplementary Material, and only summary results are included here as they relate to informing policy recommendations. While the survey probed the broader field of precision medicine, the recommendations focus specifically on genomic medicine.

## Responses to the Survey

### Knowledge About Precision Medicine

The respondents included physicians, postgraduate students, laboratory heads, scientists engaged in research activities, and an administrator. Most indicated that they were familiar with the concepts underlying precision medicine, and when asked to provide a definition of the field, the 45 individuals who answered gave reasonable responses. Over 80% indicated that they were already applying advanced genomic technologies in their research or were planning to do so in the foreseeable future. Many project descriptions agreed only marginally with the accepted definition of precision medicine, but incorporated technologies often associated with it, such as next generation sequencing (NGS). Overall, the data collected in the survey indicated that the participating scientists were cognizant of the scope of public health genomics, and that they were eager to engage in research that encompasses its premises. Since it was clear that genomic rather than precision medicine programs have been introduced in several African countries, this study focused on findings related to genomic medicine.

### Institutional Environment and Infrastructure

**FIGURE 1:**
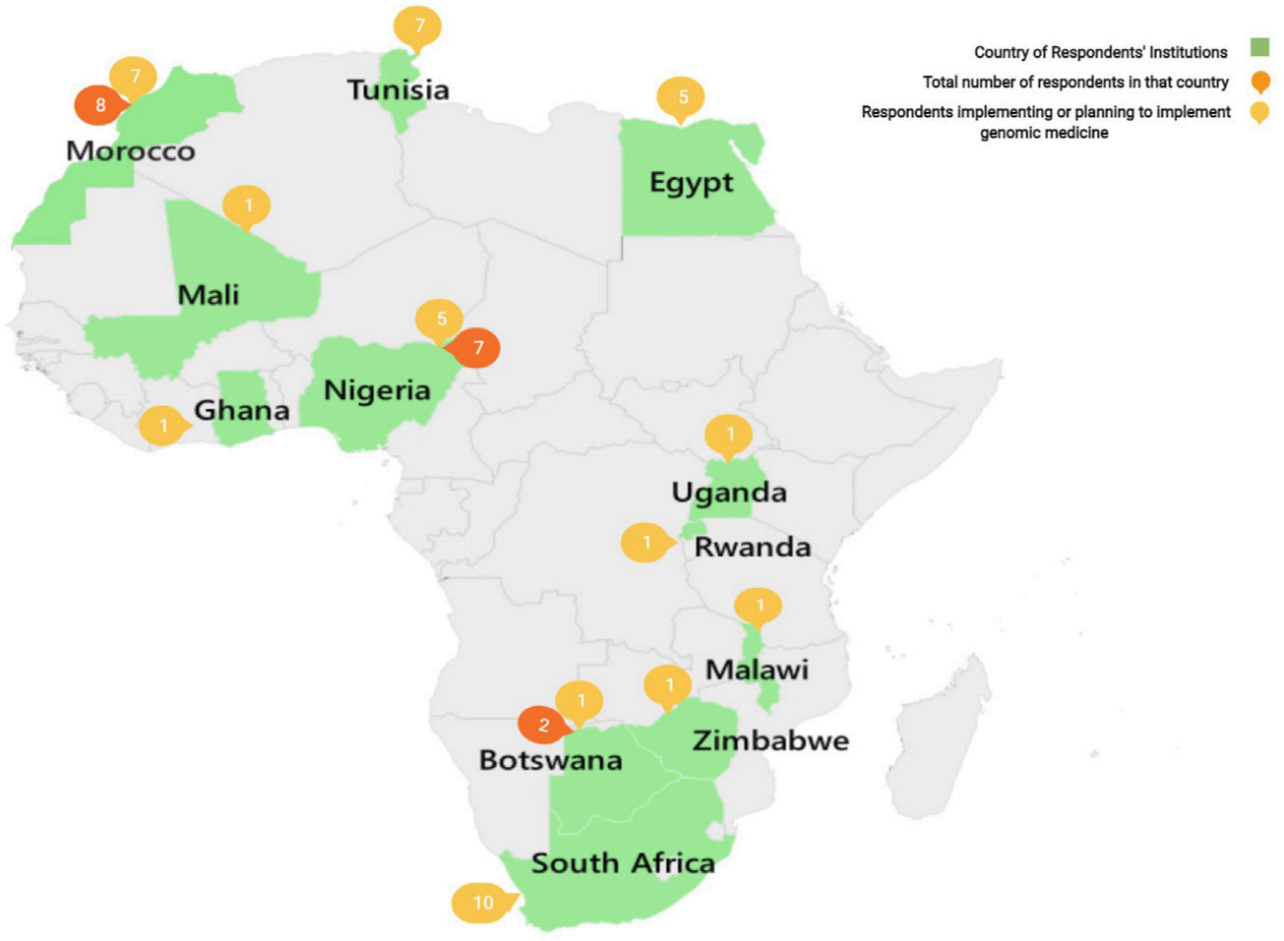
Map showing the countries from which responses were received, including the total number of respondents within the country (dark orange) and out of these, the number who are currently or planning to implement genomic medicine activities (light orange).

Respondents were from twelve African countries representing most of the Continent’s geographical areas, with the exception of Central Africa. Figure 1 shows a geographical map of Africa, indicating the sample sizes of respondents from individual countries who reported on graduate programs and core sequencing facilities supported by government laboratories, universities and other higher education institutions, and some hospitals and non-governmental organizations (NGOs). Less than half (43%) of the respondents had access to local infrastructures needed to conduct genomic medicine research, the rest either relied on external collaborators (15%) or did not have adequate access (42%). Less than half had access to a biobank (21%), a clinical laboratory (26%), a genomic analysis or sequencing facility (28%), a computational facility for data analysis (28%), or a data storage and archiving facility (26%). Very few had access to all of these essential infrastructures, suggesting that lack of access to proper infrastructure is a major roadblock to the spread of genomic medicine on the African Continent. There are, however, some state of the art research facilities in several African countries that are well placed to support genomic medicine and may be able to provide at least some of these services at a regional level.

### State of the Field and Awareness

Approximately one third of the respondents indicated that their home institution hosted a program or project focused on aspects of genomic medicine, while another third stated that there was no such program locally. The rest did not respond to this question or were unsure. The programs that were mentioned addressed a wide variety of diseases. When asked about the data that were collected on patients, only 20% of respondents stated that both demographic and clinical data were being collected. About one third of respondents collected or stored genomic/genetic data to enable sharing with other stakeholders for either research or clinical care purposes. It appears that at least five African countries (South Africa, Tunisia, Zimbabwe, Egypt, and Rwanda) have started or are planning to start national genomic medicine programs. Awareness of the field among clinicians and researchers is still limited as only 14% of respondents thought that scientists in their country were ready to implement precision/genomic medicine concepts. Most felt that either they were cognizant of the concepts but not ready to implement them (36%), or still needed to be better informed or educated (41%).

### Barriers to Implementation

Finally, the survey sought to understand the major barriers impeding the implementation of genomic medicine in African countries. The most commonly cited barrier was the lack of proper infrastructures and technologies to support research and clinical translation (81%). Limited information about determinants of disease susceptibility in local populations (64%), a lack of genetics-focused education of physicians and caregivers (67%), and a perception that this was a low-priority investment (55%) were also mentioned. Less important were the potential low-cost effectiveness (24%) and the fear that it would benefit only the most affluent patients (31%). Most respondents (83%) agreed that additional training would be required in their countries for genomic medicine to become a reality. Specialized training could target university students, house officers, physicians, genetic counselors, nurses and other caregivers, and laboratory technicians.

## Actionable Recommendations

The framework that we previously produced in the form of a policy paper (Policy Paper: A Framework for the Implementation of Genomic Medicine for Public Health in Africa | The AAS) outlined in some detail the required steps for a successful introduction of genomic medicine concepts in the African context. Due to the breadth of precision medicine and areas of expertise of the authors, the paper focused only on genomic medicine. Despite the limited sample size, the results of the survey presented in this study offer an additional window on knowledge gaps identified by respondents from twelve African countries, which should receive priority from a policy perspective as outlined below.

### Prioritization of Infrastructures

The results of the survey clearly indicate that lack of access to proper infrastructure is a major roadblock to the implementation of genomic medicine in most African countries and ensuring that such access is put in place is therefore a policy priority. The infrastructure that should be available includes simple point-of-care (POC) platforms to enable sample acquisition and accurate data capture during first-tier genetic testing; more sophisticated facilities for high-throughput data generation and analyses; biobanks for long-term storage and retrieval of patient samples; databases for demographic and clinical data capture, archiving and retrieval that can be accessed from mobile devices; and computational facilities for data analysis. Some such resources already exist in most geographical areas of the Continent, for example through the Human Heredity and Health in Africa (H3Africa) program. Specialized training in data management for return of research results is furthermore provided through the Open Genome Project (https://www.gknowmix.org/opengenome/), which explores the feasibility of combining POC genomics with genetic counselling (Oosthuizen et al., 2021). Pathology-supported genetic testing of multiple non-communicable (NCD) pathways enables genetic counselors to assess different aspects of the same disease in a patient report. Policy efforts should therefore seek to establish collaborative channels for sharing existing facilities wherever possible, and to build new ones only if absolutely necessary and in the context of a regional sharing policy.

### Need for More Translational Research

While not directly addressed in the survey, the question of whether existing scientific knowledge is sufficient to guide a path to genomic medicine in Africa is raised by many of the respondents. Most existing data on the relationships between genetic background, environmental effects and lifestyle on the susceptibility to disease, and on how to best target therapies based on detailed knowledge about a patient’s background, have been collected in populations of European or East Asian descent. The dearth of information and patient registries on such relationships among African individuals, especially in light of the high level of genetic diversity on the continent, is a major obstacle. It should therefore be an important policy objective to ensure that high quality epidemiological, clinical and omics research on the same study cohorts in their resident populations is being conducted, be it through local research initiatives or by participating in international efforts such as H3Africa, the SA-UK Newton Collaborative Research and Development Program in Precision Medicine (e.g., https://gtr.ukri.org/projects?ref=103993), or the International Hundred K + Cohorts Consortium (IHCC). There has been recent interest in establishing a longitudinal African population cohort, which would offer excellent opportunities for expanding this knowledge (https://cms.wellcome.org/sites/default/files/2021-03/APCC%20Scoping%20paper_%20FINAL_EN_0.pdf). At the very least, governments should facilitate participation of their citizens and researchers in translational research studies, and access to their health care systems, within a responsible legal framework, to enable genomic medicine.

### Information Dissemination to Potential Users

Most of the respondents to the survey thought that healthcare workers in their countries were insufficiently informed of the principles of precision medicine at the genomics level, and thus unable to participate fully in any implementation efforts. In this context, one of the respondents highlighted the benefit of positioning pathology as a bridging science at the interface between the research laboratory and clinical practice, thereby gaining collective knowledge through comparative effectiveness studies that may translate into immediate clinical benefit to patients. This suggests that informing and educating the healthcare workforce about genomic medicine and other areas of precision medicine should be prioritized before starting a national program. Existing frameworks for continuing education (e.g., clinician professional development, good clinical practice courses) should be leveraged, and targeted information campaigns (leaflets, posters, lectures, workshops, etc.) should be developed and distributed. A common theme emerging from the survey is the need to define where genomic tests add value to standard pathology routinely applied in precision medicine.

### Training Programs for Specialized Personnel

Successful implementation of a precision medicine program will require a new workforce with updated skills, able to respond to the challenges associated with the implementation. This requires upskilling of existing workers and adjustment of current curricula to produce an interdisciplinary workforce. This is especially true in Africa, where training is often focused on technologies in current use rather than new, leading-edge knowledge; and this is exacerbated where resources for new technologies are not yet in place. Lack of a highly trained specialized workforce as a significant obstacle was clearly described by respondents. The list of required specialties is extensive: researchers in genetics, omics, statisticians, genetic/genomic counselors, bioinformaticians, computer systems administrators, engineers capable of maintaining sophisticated instruments and technicians to operate them. There is also a deficit in soft skills including leadership, management, and governance. The genomics innovation ecosystem should therefore engage with clinicians, patients, researchers, service providers, data curators, data consumers, funding agencies, pharmaceutical and technical/manufacturing companies, policymakers, medical insurance companies, civil society, learned societies, etc., All these stakeholders should acquire specific skills and require specific training activities. While it may be necessary to create some training programs from scratch (including medical scientist professional development courses and relevant degree programs), the need for specialized personnel can probably be largely met by updating curricula in existing educational institutions and encouraging students to pursue training in these new fields. There should be a clearly stated policy encouraging institutions to update their curricula and to train the required workforce in advance or in parallel to putting in place genomic medicine and genetic pathology programs that will safeguard personal information processing. Due consideration should be given to leveraging training opportunities provided by the Wellcome Connecting Science, H3Africa and others offered in-country by African scientists.

### Engage Political Stakeholders and the Public

Successful implementation of genomic medicine approaches in healthcare systems critically depends on the engagement of knowledgeable politicians, who in turn will need the support of their voters. Genomic programs should be tightly integrated with existing governmental health and legal systems. Also, in the absence of understanding and support from the medical profession and the general public, uptake will be limited, and such programs are more likely to fail. Themes to be highlighted include: how each patient will receive care better adapted to their personal circumstances; how more emphasis will be given to prevention of advanced disease; how treatments that are not efficacious will be avoided, and that the overall cost of providing such care will be less. It should also be emphasized that everyone will benefit, not only the privileged classes, and that population-level research using genomic medicine approaches can also ensure general improvements to health care provided to specific communities. Leaders of genomic programs should ensure that from the start they engage policy makers and the general public, not only to provide financial and logistical means, but also to ensure broad popular support for the initiatives and to proactively address questions and concerns raised by the general public and healthcare clients.

## Implementation Strategy

Translation of evidence-based guidelines to health practice is one of the most challenging aspects of applied research and genetic testing service delivery, especially in resource-limited settings (Mitropoulos et al., 2015; Kristensen et al., 2016; Fontes Marx et al., 2021). Implementation of genomic medicine requires a dynamic process informed by the nature of the innovation and the manner in which the type and strength of the evidence is communicated to potential users. The slow pace of adoption of novel findings largely relates to poor understanding of the role of genomics in risk stratification and targeted treatment. Practice-changing solutions require inventive steps which demonstrate clinical utility beyond standard pathology tests, as evidenced in the surgical oncology field using a cost-minimization pathology-supported genetic testing strategy (Mampunye et al., 2021; Myburgh et al., 2021). Incorporation of omics solutions into existing treatment algorithms is therefore needed to prevent misdirection and fragmentation of genetic services relevant to individual patients or their healthy, at-risk family members (Solomon et al., 2011; Moremi et al., 2021). This may involve molecular profiling of not only a few genes, but also whole exomes and genomes in uninformative cases. Highly dense datasets are therefore becoming the norm for disease screening, risk stratification and therapeutic interventions.

Important aspects to consider for successful implementation of genomic medicine and policy development in African countries, are summarized in Table 1. This information is partly based on the pathology-supported genetic testing method being introduced as a case study across the illness and wellness domains (Okunola et al., 2019; Moremi et al., 2021). The list of requirements for implementation of genomic medicine in clinical care was informed by our survey, the previously mentioned policy framework compiled by 38 African scientists, as well as insights gained from literature studies and genome-scale sequencing recently performed in African facilities (Torrorey-Sawe et al., 2020; Glanzmann et al., 2021). The checklist provided in Table 1 may be used to guide the process from scientific discovery to clinical application, while accommodating infrastructure barriers and knowledge gaps that may be encountered in the research translation process.

**TABLE 1:** Checklist with key requirements to assess readiness of African countries prior to implementation of genomic medicine programs.

Key elements	Processes required	Readiness assessment
Patient selection: Clinical facilities for patient counselling, screening, treatment and monitoring	Informed consent of participants, obtain relevant previous pathology/other test results from health records, data translation into an adaptable report, genetic counselling	Clinical infrastructure for patient enrollment, collection and analysis of biosamples linked to patient data to enable treatment recommendations and monitoring of clinical outcome
Sample selection: Sample collection, processing and storage facilities, data acquisition tools to prevent operational fragmentation	Sample type selection (e.g., blood, saliva, biopsy) and metadata collection, sample transfer and preparation applying good clinical practice	Biorepositories for sample preparation and storage to enable retrieval and re-analysis of patient samples, acquisition and storage of metadata including clinical data from different sources
Data generation: Genetic testing, genomics data generation and storage	DNA/RNA extraction or direct swab-specimen application, quality control, data generation through genetic testing or omics technologies	Data generation instruments for generation of results on portable devices (point-of-care/other genotyping tests) and/or large scale (microarrays, high-throughput sequencing)
Data analysis: Data storage, curation, analysis and interpretation by assessing clinical relevance of genetic findings	Data processing, analysis, variant classification, identification of actionable gene variant(s), analytical validation using gold standard methodology, alignment of clinical characteristics with familial vs. lifestyle risk and/or treatment response	Data and computing infrastructures for acquiring and storage of genomic data and to enable efficient and secure transfer of data, complex software environment for running research-informed pipelines to enable analysis and interpretation of high throughput genomic data, integrated data systems for analysis, interpretation and report generation, and AI to facilitate clinical decision making
Knowledge databases: Up to date information on genotype-phenotype links and evidence for actionability	Compare variants with reference and disease datasets and prior evidence, extract additional clinically relevant data (e.g., medication use, comorbidities) for clinical interpretation	Reference genomics datasets for relevant populations, sufficient evidence for actionability based on data generated in-country and reported in other patients with similar clinical phenotypes based on well-established scientific literature
Research facilities: Increase knowledge on genomics in African populations, enable translation of results	Data generation and sharing for research translation, gain collective knowledge for precision medicine applications, validation and transition to clinical application	Data generation and sharing capability, research infrastructure, facilities for transitioning to applied research involving feasibility and proof of principle studies on assay validation, clinical utility and health economics to enable up-scaling for clinical translation and implementation
Training: Genomic medicine training programs for healthcare professionals and support personnel	Training to develop a multi-disciplinary service delivery team, case-based learning to achieve learning objectives	Training facilities and curricula for new degrees and professional development courses, online platforms to practice implementation ideas (e.g. pathology-supported genomics)
Regulatory framework: Policy development for governing relevant activities, informed by standard operating procedures (SOPs) and instructions for use (IFUs)	Data and sample governance policy, informed consent documents and tracking, material transfer and data sharing agreements, participant engagement and intellectual property disclosure to authorities	Genomic medicine framework with data and sample governance, and ethical oversight, long-term participant engagement to close the gap between expectation and reality

Molecular genetic testing has raised a variety of policy issues (Klein 2020), ranging from privacy to reimbursement challenges, including the development of medical insurance coverage policies for genetic tests. For regulatory approval of laboratory-developed genomic solutions, we need to leverage the strengths of both precision medicine and public health genomics (Roberts et al., 2021). Moving from basic research to translation requires effective management of the genomic service delivery process from sample collection to report generation, which should fit seamlessly into existing clinical workflows and treatment algorithms. Given the potential of biochemical methods and other health assessments to uncover the genetic component of complex, multifactorial diseases in a clinical context, we envisage a future where research databases (e.g., https://www.cansa.org.za/establishing-cancer-genomics-registry-to-support-implementation-of-personalised-medicine) are developed in parallel with patient care for differential diagnosis and long-term pharmacogenomics informed clinical outcome studies. Use of the multi-assay pathology-supported genetic testing platform towards seamless integration of research and service delivery has recently been recognized as best practice by the International Consortium of Personalized Medicine (ICPerMED).

## Conclusion

This policy brief highlights important requirements for successfully implementing genomic medicine programs in Africa. The survey that we conducted for feedback from stakeholders about their perceptions on this healthcare model was limited by sample size and lack of broad representation across sectors. However, the results we did receive from multiple institutions in twelve countries of varying economic levels (low- and middle-income countries), as well as our broader effort to outline the challenges faced when putting in place genomic medicine programs, have allowed us to formulate reasonably actionable recommendations that are broadly applicable. The finding that only 10% of scientist respondents were ready to or have already started a process of implementing genomic medicine in their country was concerning. This led to inclusion of a checklist (Table 1) as an educational tool to assist scientist entrepreneurs with identification of infrastructural and other gaps they need to address in their translational research projects. The pathology-supported genetic testing strategy, for example, makes use of a dynamic screening algorithm for eligibility assessment and clinical interpretation of genomic tests by guiding treatment decision-making beyond a single objective. The policy recommendations we provide would not necessitate major investments, although aspects such as data sharing and integration platforms may require stronger cross-border cooperation and joint investments than are currently ongoing in the region. Broad buy-in from healthcare providers, educational institutions, and government services as well as coordinated activities between them will be key to success.

## Data Availability

The datasets presented in this article are not readily available because the raw results of the survey cannot be shared to protect the privacy of participants. Summary results are available in the Supplementary Material.

## References

[B1] Fontes Marx M. Ataguba J. E. Vries J. D. Wonkam A. (2021). Systematic Review of the Economic Evaluation of Returning Incidental Findings in Genomic Research. Front. Public Health 9, 697381. 10.3389/fpubh.2021.697381 34277554 PMC8281014

[B2] Glanzmann B. Jooste T. Ghoor S. Gordon R. Mia R. Mao J. (2021). Human Whole Genome Sequencing in South Africa. Sci. Rep. 11, 606. 10.1038/s41598-020-79794-x 33436733 PMC7803990

[B3] Klein R. D. (2020). Current Policy Challenges in Genomic Medicine. Clin. Chem. 66 (1), 61–67. 10.1373/clinchem.2019.308775 31699701

[B4] Kristensen N. Nymann C. Konradsen H. (2016). Implementing Research Results in Clinical Practice- the Experiences of Healthcare Professionals. BMC Health Serv. Res. 16, 48. 10.1186/s12913-016-1292-y 26860594 PMC4748469

[B5] Mampunye L. Grant K. A. Peeters A. V. Torrorey-Sawe R. French D. J. Moremi K. E. (2021). MammaPrint Risk Score Distribution in South African Breast Cancer Patients with the Pathogenic *BRCA2* c.7934delG Founder Variant: Towards Application of Genomic Medicine at the Point-of-Care. Breast 56 (Suppl. 1), S17–S90. 10.1016/s0960-9776(21)00120-x

[B6] Mitropoulos K. Al Jaibeji H. Forero D. A. Laissue P. Wonkam A. Lopez-Correa C. (2015). Success Stories in Genomic Medicine from Resource-Limited Countries. Hum. Genomics 9 (1), 11. 10.1186/s40246-015-0033-3 26081768 PMC4485996

[B7] Molster C. M. Bowman F. L. Bilkey G. A. Cho A. S. Burns B. L. Nowak K. J. (2018). The Evolution of Public Health Genomics: Exploring its Past, Present, and Future. Front. Public Health 6, 247. 10.3389/fpubh.2018.00247 30234091 PMC6131666

[B8] Moremi K. E. Scott C. J. de Jager L. J. Pienaar R. Zemlin A. E. Kotze M. J. (2021). Implementation of a Pathology-Supported Genetic Testing Framework for Return of Research Results to Family Members of Deceased Breast Cancer Patients with Somatic *TP53* Variants. The Breast 56 (Suppl. 1), S64–S65. 10.1016/s0960-9776(21)00205-8

[B9] Myburgh E. J. de Jager J. J. Murray E. Grant K. A. Kotze M. J. de Klerk H. (2021). The Cost Impact of Unselective vs Selective MammaPrint Testing in Early-Stage Breast Cancer in Southern Africa. The Breast 59, 87–93. ISSN 0960-9776. 10.1016/j.breast.2021.05.010 34217105 PMC8259301

[B10] Okunola A. Torrorey-Sawe R. Baatjes K. J. Zemlin A. E. Erasmus R. T. Kotze M. J. (2019). Investigation of the Role of Vitamin D Metabolism in South African Breast Cancer Patients Using Whole Exome Sequencing. The Breast 44 (Suppl. 1), S36. 10.1016/s0960-9776(19)30162-6

[B11] Oosthuizen J. Kotze M. J. Van Der Merwe N. Myburgh E. J. Bester P. van der Merwe N. C. (2021). Globally Rare BRCA2 Variants with Founder Haplotypes in the South African Population: Implications for Point-of-Care Testing Based on a Single-Institution BRCA1/2 Next-Generation Sequencing Study. Front. Oncol. 10, 619469. 10.3389/fonc.2020.619469 33643918 PMC7908826

[B12] Roberts M. C. Fohner A. E. Landry L. Olstad D. L. Smit A. K. Turbitt E. (2021). Advancing Precision Public Health Using Human Genomics: Examples from the Field and Future Research Opportunities. Genome Med. 13 (1), 97. 10.1186/s13073-021-00911-0 34074326 PMC8168000

[B13] Solomon B. D. Pineda-Alvarez D. E. Hadley D. W. Teer J. K. Cherukuri P. F. Hansen N. F. (2011). Personalized Genomic Medicine: Lessons from the Exome. Mol. Genet. Metab. 104 (1-2), 189–191. 10.1016/j.ymgme.2011.06.022 21767969 PMC3171610

[B14] Torrorey-Sawe R. van der Merwe N. Mining S. K. Kotze M. J. (2020). Pioneering Informed Consent for Return of Research Results to Breast Cancer Patients Facing Barriers to Implementation of Genomic Medicine: The Kenyan BRCA1/2 Testing Experience Using Whole Exome Sequencing. Front. Genet. 11 (170), 170–176. 10.3389/fgene.2020.00170 32231682 PMC7089032

