# Supplementary material for: A View on Genomic Medicine Activities in Africa: Implications for Policy
Source: Front Genet. 2022 Apr 27;13:769919. doi: 10.3389/fgene.2022.769919 (PMC9091728; doi:10.3389/fgene.2022.769919)
Supplement: Supplementary file 1 [file DataSheet2.PDF]

African Precision Medicine survey response report

Background

One of the tasks assigned to the African Precision Medicine Task Force was to assess the state of awareness and extent of penetration of precision medicine on the African continent. To this effect, a questionnaire was created that probed five overall areas: (A) knowledge of the respondent about Precision Medicine (PM); (B) institutional environment and infrastructure; (C) implementation in the respondent's institution; (D) awareness of precision medicine in the respondent's country; and (E) potential obstacles and opportunities. The hope was that the responses to the questionnaire would help inform appropriate approaches to the implementation of precision medicine in various parts of the African continent. Although the number of complete responses was relatively small, information obtained is considered valuable to provide an overview of the current state of precision medicine in Africa and supports the implementation plan for public health genomics recommended in the Policy Paper published in February 2021.

Methods

An online questionnaire with 25 questions encompassing the areas outlined above was created by the Task Force and implemented using REDCap. The full questionnaire is available at http://j.mp/37YDNuw. A request to complete the questionnaire was sent to the members of the H3Africa Steering committee, to the H3ABioNet network, as well as to over thirty African stakeholders and disseminated more widely via mailing lists and social media. The respondents were also asked to pass the questionnaire along to their colleagues. A period of six weeks was allowed to collect responses. The responses were exported and summarized in an Excel spreadsheet. Incomplete responses were manually curated and removed from the survey results. Given the relatively small number of valid responses, a qualitative analysis was performed to gain insight from African scientists actively involved in genomic research and/or service delivery.

Results

A total of 78 responses were received, of which 55 were complete enough to be included in the analysis. The incomplete responses included completely void entries, duplicates, and entries with names and emails only. All of these were removed. The results are presented across five areas numbered from A to E, with the individual questions addressed under each of these sections.

A. RESPONDENT'S KNOWLEDGE ABOUT PRECISION MEDICINE

Your current position?

A total of 54 participants responded to this question, some of which provided multiple responses. As shown in Figure 1 most (37) respondents identified themselves as research scientists, including graduate students (11), clinicians (10), and division heads (8), with one administrator and 11 "others" mostly engaged in research. The sample of respondents is therefore reasonably representative of different positions.

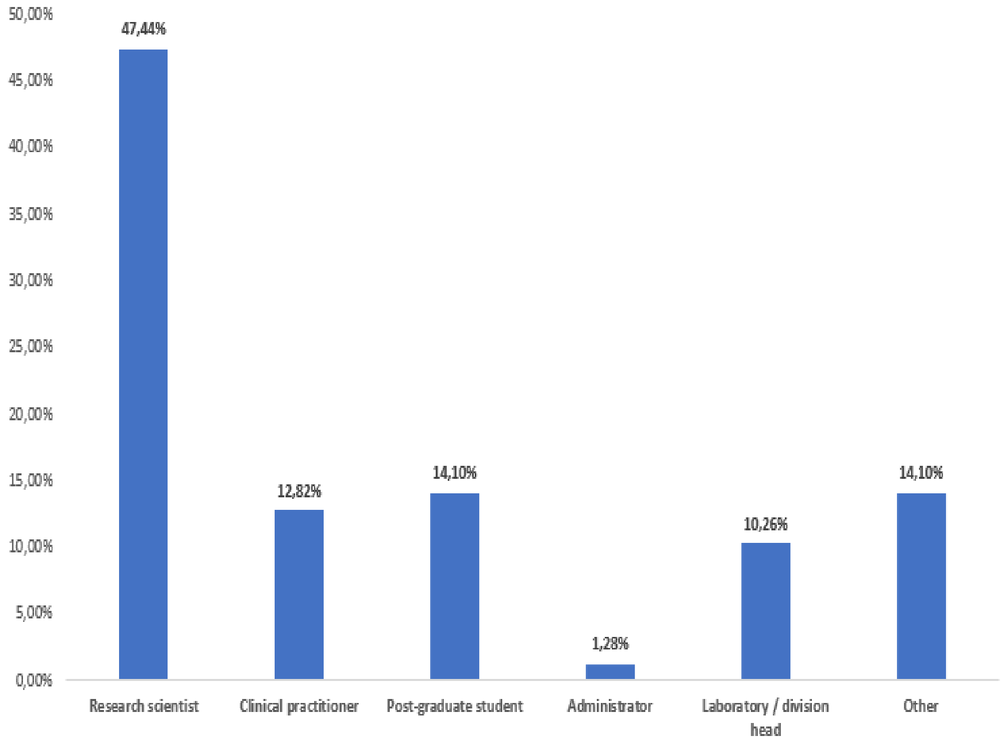

Figure 1: Current role in your institution

How familiar are you with the concepts of Precision Medicine?

The respondents were, on average, rather conversant with the concepts, with an average self-described score of 64/100 (Figure 2).

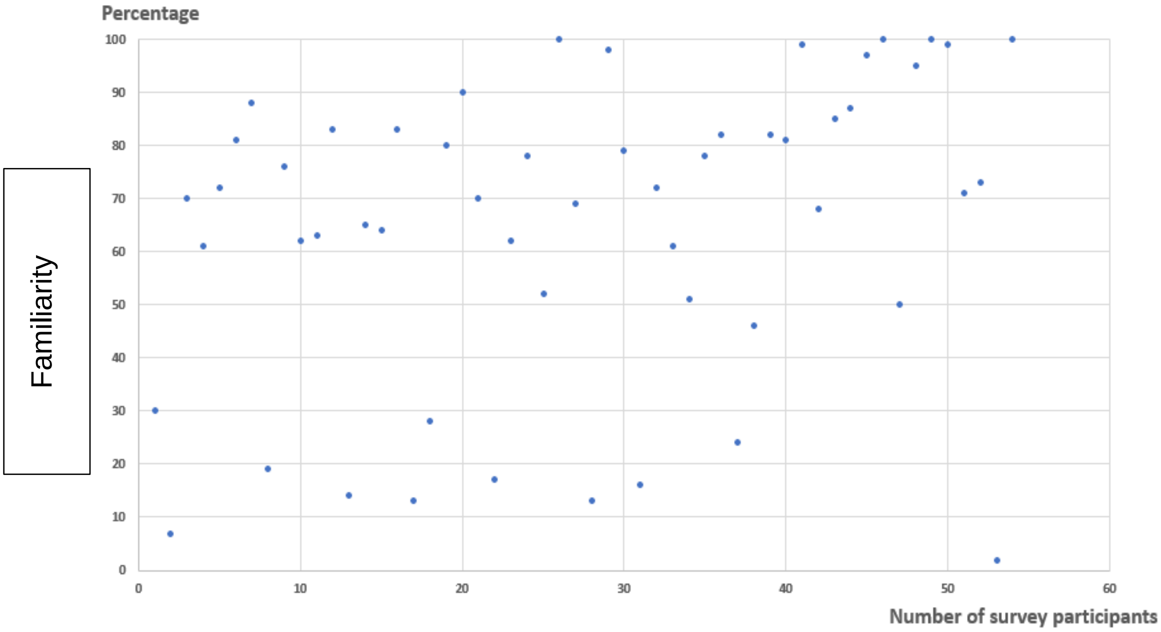

Figure 2: Participants' familiarity level with the Precision Medicine concepts

How would you define 'Precision Medicine'?

Out of 55 responders, 10 did not give an answer. Most of the others (n=7), with a few exceptions, gave reasonable answers, showing at least passing familiarity with the concept.

Are you now, or are you planning to use Precision Medicine approaches in your work?

As shown in Figure 3, 23 respondents already used PM approaches at the time of the survey, 24 were planning to do it in the future, and 7 were not planning to do it (one response was left blank).

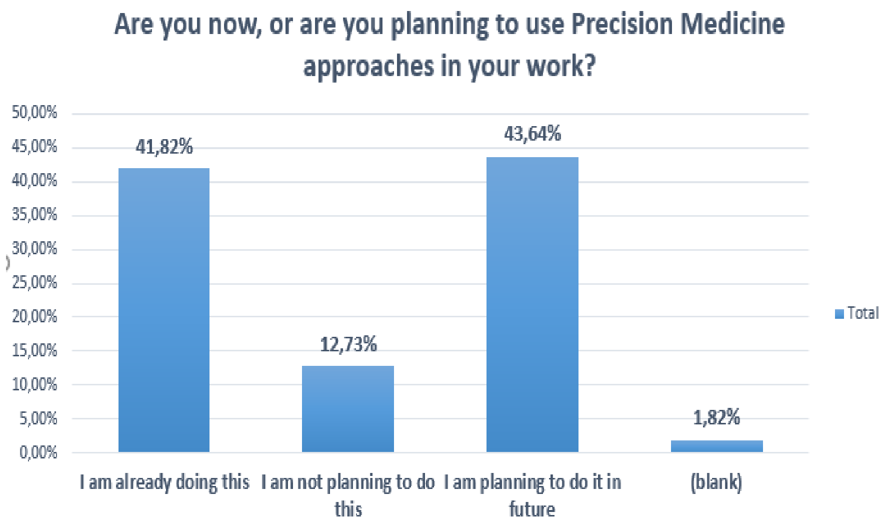

Figure 3: Use of Precision Medicine approaches

If you responded positively to the previous question (23 responded positively), please describe briefly your Precision Medicine activities

Twelve respondents gave no answer, mostly because they do not currently have activities in this area. Responses from the rest describe a wide variety of research projects with varying relevance to the implementation of PM, from basic research to data analysis methods, to elucidation of the genetic determinants of diseases.

B. INSTITUTIONAL ENVIRONMENT AND INFRASTRUCTURE

In what country is your institution located?

There were 44 responses from 12 African countries, and one from the USA (Figure 4). Countries represented were Tunisia 13% (7), Egypt 9% (5) and Morocco 13% (7) (North Africa); Nigeria 2% (6), Mali 2% (1) and Ghana 2 % (1) (West Africa); Rwanda 2 %(1), Uganda 2 %(1) Malawi 2% (1) (East Africa); Zimbabwe 2% (1), Botswana 4% (2), and South Africa 18% (10) (Southern Africa).

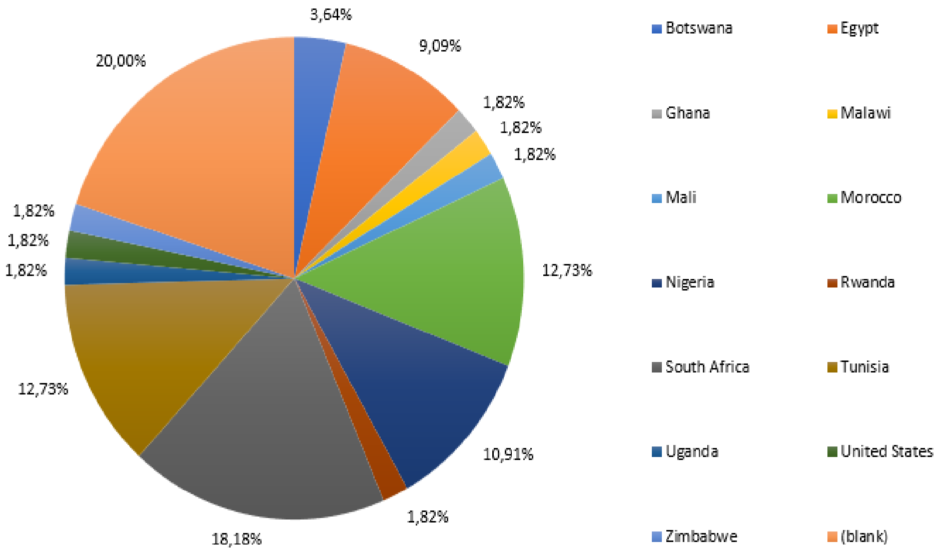

Figure 4: Country of location of respondents

What type of institution is it?

Out of 44 responses depicted in Figure 5, 10 were government research institutions, 26 were universities or technical colleges, 2 were hospitals or clinics, 3 were independent organisations, and 3 were NGOs.

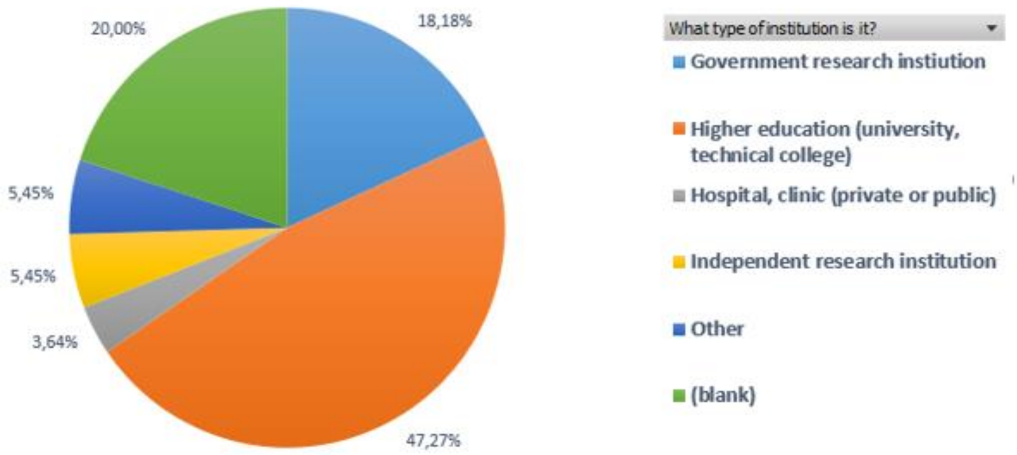

Figure 5: Type of Institution

Do you have access to the required infrastructures for your Precision Medicine work?

Out of the 53 responses shown in Figure 6, 20 respondents had no access (36%), 8 had access through external collaborations (15%), and 25 had access at their local institutions (46%).

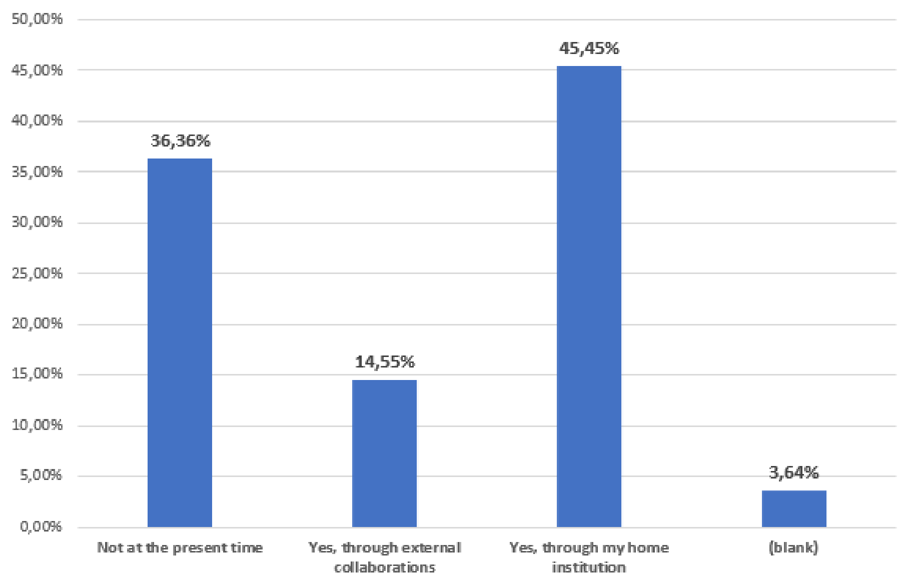

Figure 6: Institutions' access to the required infrastructures for Precision Medicine

To what infrastructures does your institution have access?

Fifty four participants responded to this question, many of whom provided multiple responses. Nineteen had access to a biobank, 25 to a standard clinical laboratory, 36 to a research laboratory, 27 to a genomic analysis facility, 26 to a computational facility for data analysis, and 24 to a data storage and archiving facility. It is noteworthy that on average any single type of facility other than a research laboratory was available for only half the respondents.

C. FOCUS AREAS AND DATA COLLECTION

What is the primary research focus of your group or department?

The responses revealed that participating institutions in the survey are focusing on diverse research topics, with a few specializing in different disease types with interest in precision medicine (Figure 7).

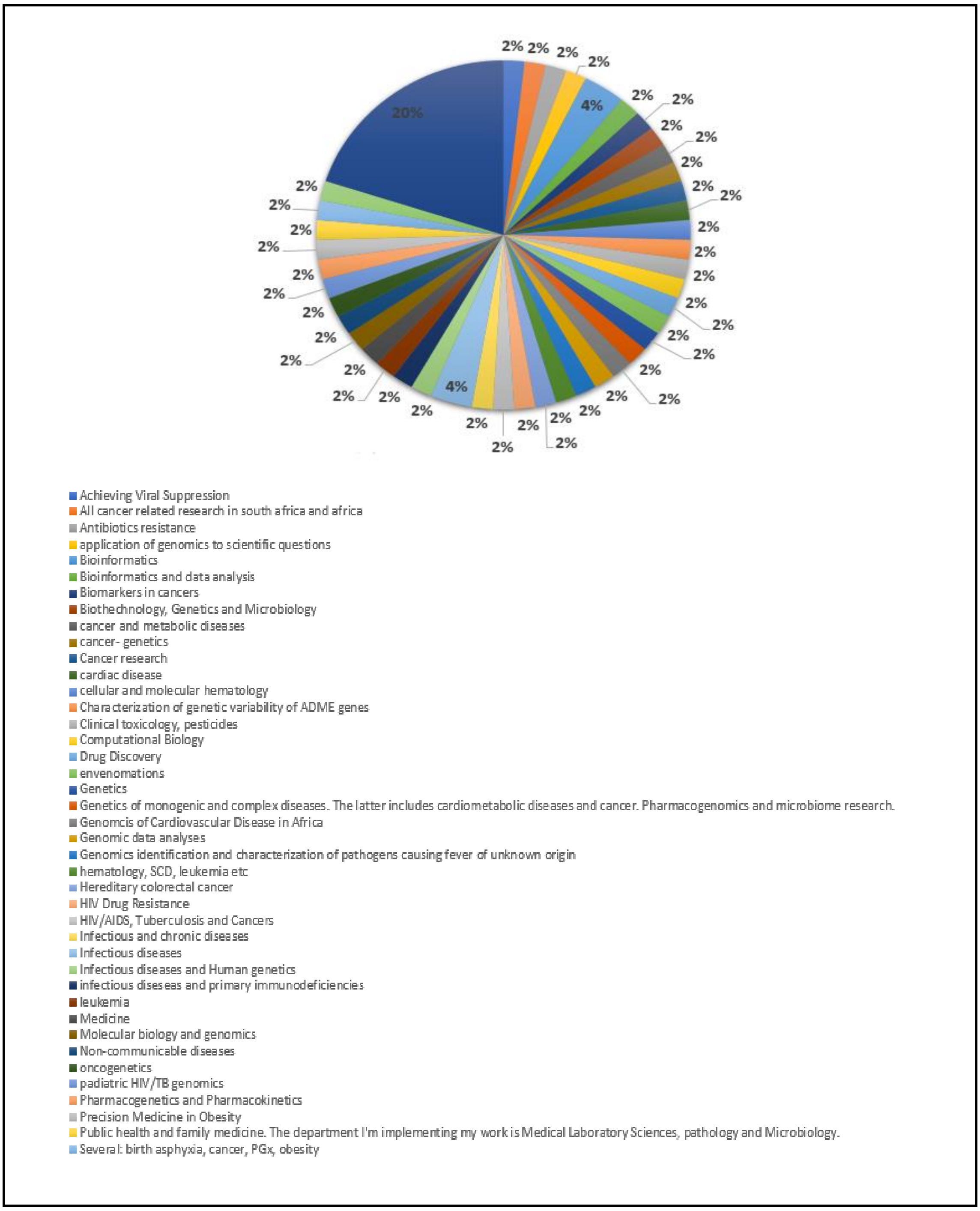

Figure 7: Groups' primary research

Does your institution have a Precision Medicine program (clinical or research)?

The results showed that 31% declared having a Precision Medicine program, the remaining either don't (31%) or are not aware of the existence of one (18%) (Figure 8).

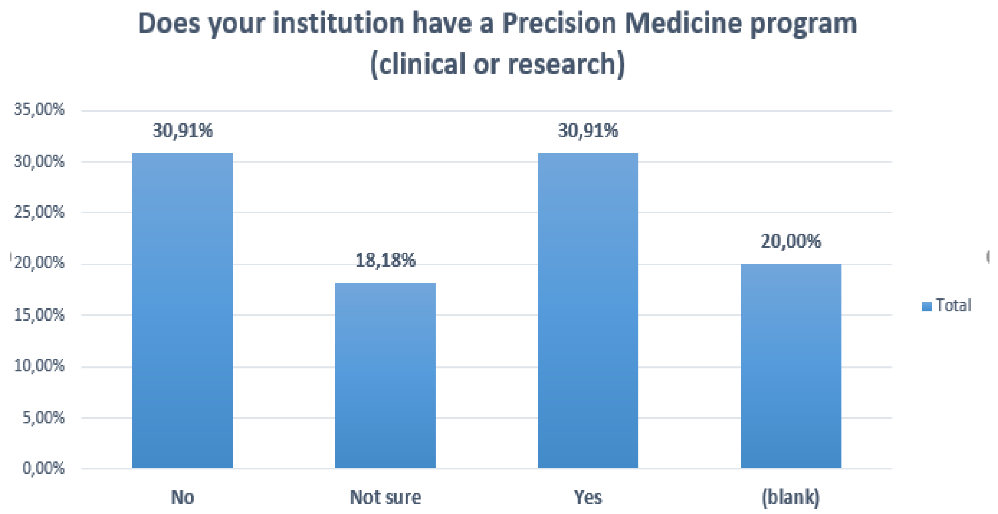

Figure 8: Availability of institutional precision medicine program (clinical or research)

If your institution has a Precision Medicine program, which diseases or patient populations does it target?

The responses revealed various programs within the African institutions, which have Precision Medicine programs ranging from clinical (Cancer, Infectious diseases, Neurological diseases, Genetics) to educational programs.

If your laboratory or group collects data about patients, which of the following statements apply?

Figure 9 shows the different types of data collected by respondents, including demographic, clinical and genomic data. In addition, the ethics and sharing processes for the collection are independently reviewed and approved for almost all participants.

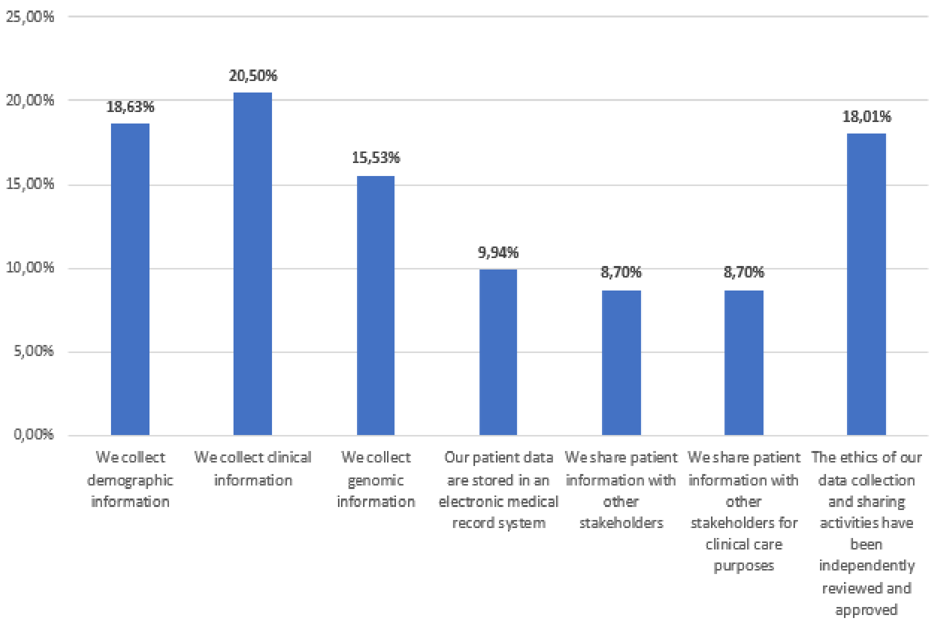

Figure 9: Types of data collected

To what infrastructures and technologies does your institution have access?

The majority of participants indicated having access to various infrastructures, from collecting samples and storing them in clinical laboratories and bio-banks, to research laboratories and genomic analysis facilities and computational facilities for data analysis and storage (Figure 10). The access to these infrastructures and facilities is not at the same level and was noted as a challenge for most participants.

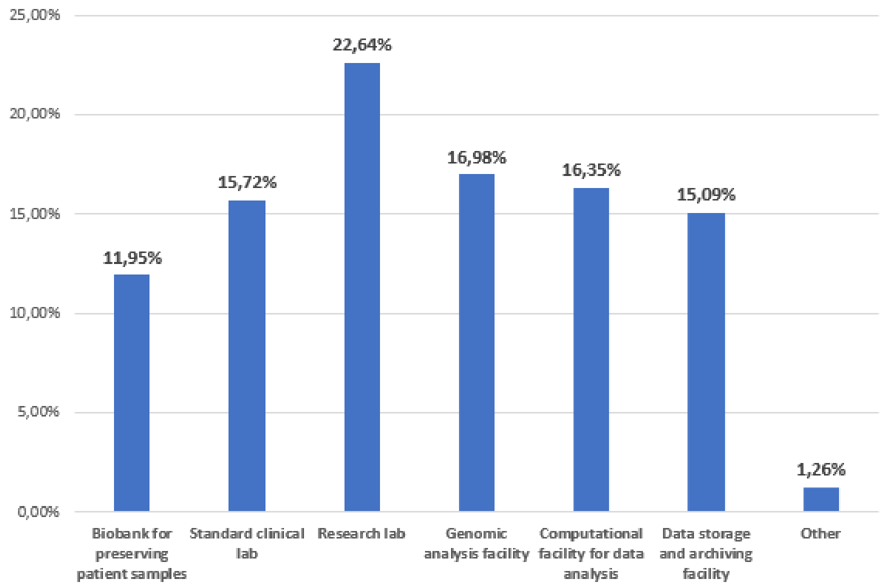

Figure 10: infrastructures and technologies that institution have access to

D. AWARENESS

What is the level of awareness of Precision Medicine in your country?

The type of responses (Figure 11) received in this section of the survey raised the question of whether the findings reflect a real or perceived awareness among respondents. For appropriate interpretation of the results, it is important to know whether the answers reflected in figures 11, 12 and 13 are based on inventive steps taken in the respective countries to implement precision or genomic medicine in Africa, beyond data and knowledge generation. Therefore, extension of this survey by asking respondents for information on which `their answers were based would have been useful, if possible, in future. Examples are clinician education courses, impact of published research findings resulting from Government or other investment programmes, or the ethical frameworks put in place to govern responsible use of clinically-enriched genetic data for possibly addressing different aspects of the same disease in a patient. Implementation of precision medicine is a complex, incremental process that requires evidence of analytical and clinical validation of tests as well as proof of clinical utility. In this context, it is important to note that many pathology tests used in routine clinical practice do not fulfill all three criteria. This provides an opportunity to incorporate genetic testing at critical control points in the clinical pipeline, with the aim to overcome the limitations of individual health disciplines when used in isolation.

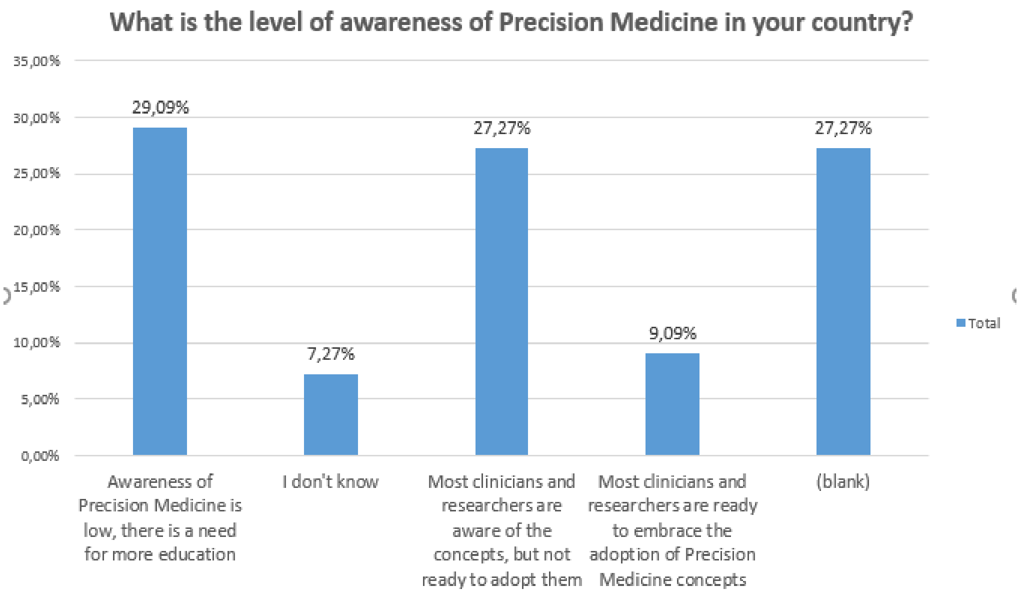

Figure 11: Level of awareness of Precision Medicine in participant's countries

Is your Government investing in a Precision Medicine program or planning for it?

The answers varied, with most answering "not sure" (Figure 12). The 10 "yes" answers received from respondents in African countries were from South Africa, Tunisia, Zimbabwe, Egypt and Rwanda.

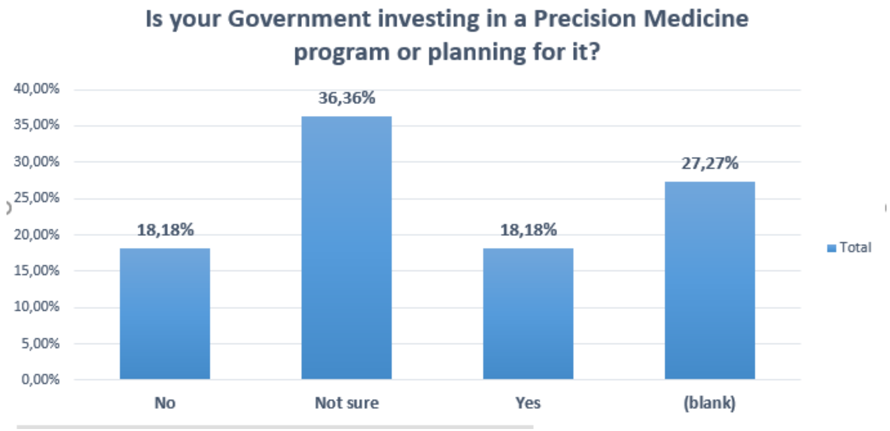

Figure 12: African governments investment in Precision Medicine

If so, could you tell us more about the scope of this program and who is running it?

The following responses were obtained in relation to academic and government funded initiatives:

- The Ministry of Higher Education in Egypt opened a call in 2020 for Precision Medicine Network Development of Scientists that are working in the field.
- Through the South African Medical Research Council (SAMRC), the South African Government is investing in research toward Precision Medicine. A Human Genome Centre was established in collaboration with the Beijing Genome Institute for sequencing of whole human genomes. There is also a national research infrastructure programme referred to as DIPLOMICS and the

Government supported the Southern African Human Genome Programme, with the aim to improve the health of populations through genomics. The latter is run by a small group of academic researchers.

- The Strategic Health Innovation Partnership Program is run by the SAMRC with a strong focus on Precision Medicine. The South African Government is also investing in Precision Medicine through the Technology Innovation Agency supporting the Open Genome Project focused on the development of an adaptable report template for whole exome/genome sequencing.
- The Department of Science and Innovation in South Africa is spearheading several initiatives in the country to increase awareness, increase translation and promote commercialization.
- The Ministry of Higher and Tertiary Education, Science and Technology development (MHTESTD) has identified Genomics as an area of national strategic importance and had it approved by Cabinet. The Ministry has gone on to approve the establishment of the Zimbabwe Genomic Centre and provided seed funding for its construction.
- The Tunisian Ministry of health is running a big project on precision Medicine, called GENOME TUNISIA.

Are there any institutions in your country that are implementing Precision Medicine approaches?

As shown in Figure 13, the majority of respondents indicated that they do not know of institutions in their country that are implementing PM. The highest activity was reported in Research Institutions. Public and private hospitals are implementing PM approaches to a similar extent (12-14%), with the lowest uptake by individual physicians/other.

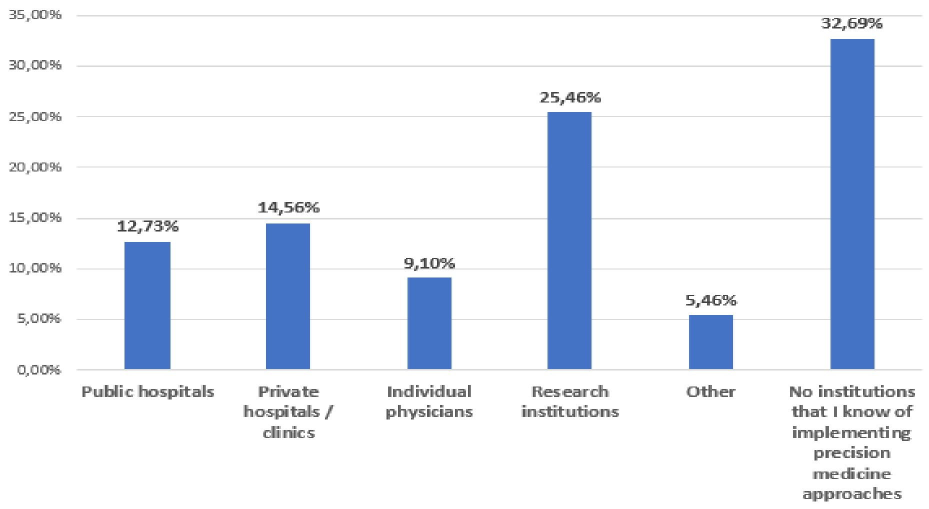

Figure 13: Types of Institutions implementing Precision Medicine approaches

E. BARRIERS AND WAYS TO OVERCOME THEM

What are the barriers to implementing Precision Medicine in your country?

The barriers encountered were diverse (Figure 14); the most common was the lack of supporting infrastructures and/or technologies (25.40%), second was both the lack of information about determinants of diseases susceptibility and the lack of education of physicians, researchers and care givers (19.84%); followed by the perception that this is a low priority investment for improvement of healthcare (17.46%) and last we have the fear that this will benefit only a small proportion of the population (9.52) and that it is not cost effective (7.94).

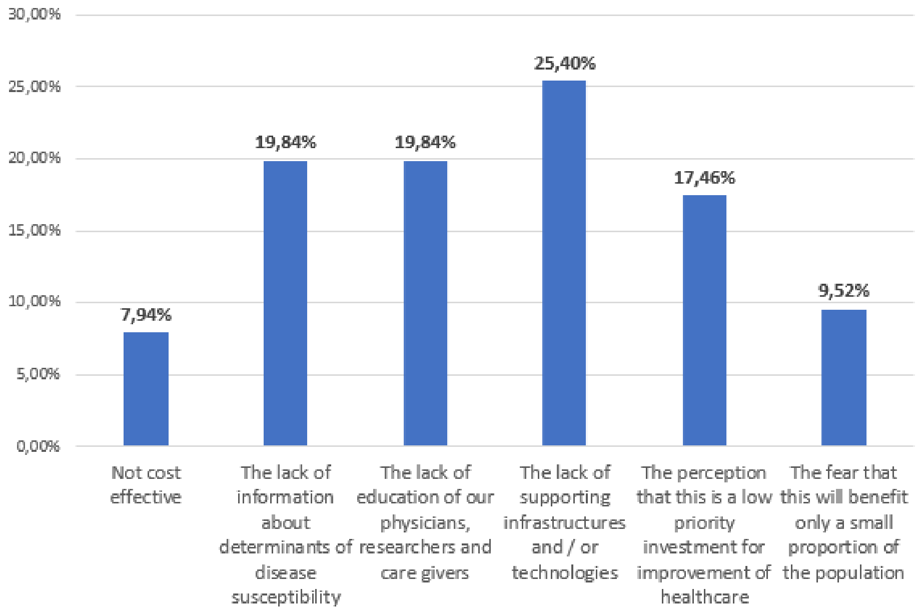

Figure 14: Barriers to implementing Precision Medicine

Other barriers?

For this question, we received only two responses as shown below:

- Ever changing landscape of genomics, differences in opinion on the value added and level of evidence required to apply PM based on available information
- Cost

In your opinion, what additional training would be required in your country to start a Precision Medicine program?

Figure 15 shows the different avenues of further training explored among survey respondents. A formal degree program for physicians was expressed as the most pressing need (20.5%), followed by genetic counsellors and laboratory technicians (both 18.6%), nurses and primary care givers (14.3%). Computational (15.5%) and specialized training abroad (12.4%) were also supported by respondents.

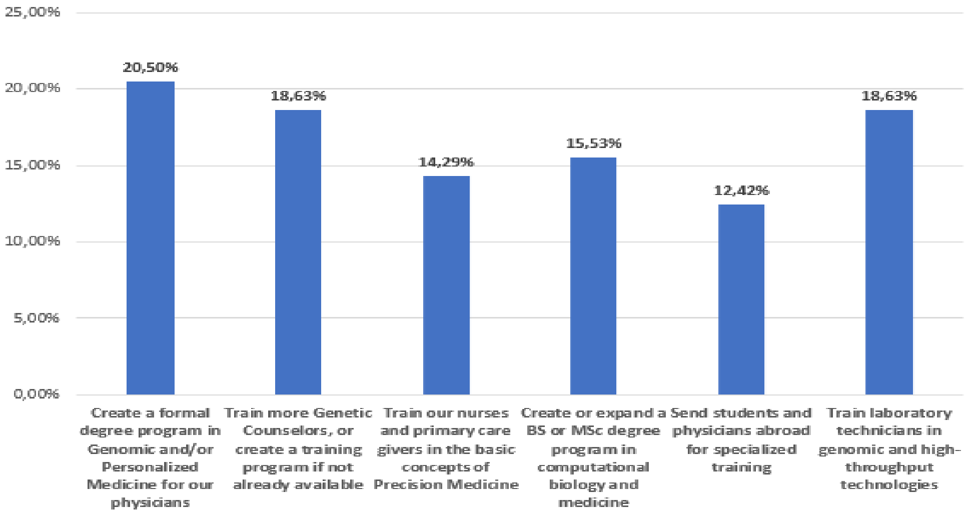

Figure 15: Additional training would be required in your country to start a Precision Medicine program

Other additional training?

Among all participants, only one provided response regarding the question about other additional needed training. The response was that case based learning provided by clinicians already applying the concepts of precision medicine should be encouraged.

F. Interpretation and Conclusion

Through this survey, precision medicine activities and awareness among African health structures and institutions were explored to help define the way forward. Our objectives were not only to explore competency levels and to assess the state of awareness among African respondents, but also to identify existing appropriate infrastructures and training needs of Precision Medicine on the African continent. Insight gained from the level of knowledge and awareness evident from inventive steps already taken by a number of respondents, should be applied to turn existing obstacles into new opportunities explored by research groups striving to achieve unique goals in their environment.

We received 55 usable responses of which 15 were only partially completed. The low response rate may be due to inadequate distribution of the survey and lack of interest/readiness for adoption of precision medicine in clinical practice. The request to participate in the survey may also have been ignored due to "information overload" or limited knowledge about this emerging healthcare model. Despite these limitations, the collected responses improved our understanding of the reasons for the lack of PM applications in some African countries and institutions. Analysis of the data revealed that knowledge about precision medicine is scarce and disparate, though most of the respondents, with a few exceptions, gave reasonable responses showing at least passing familiarity with the PM concept. It is evident that the infrastructure is still limited and specialized training is required to develop competencies and pull together different aspects of precision medicine.

Discrepancies were revealed at all levels relevant to the implementation of precision medicine in Africa, which should be addressed to improve the healthcare of African populations. To succeed with implementation of precision medicine in Africa, commitment is required from African researchers and clinicians working closely with their governments, and to expand collaboration between African and non-African countries within the framework of an African

precision medicine network. The results of this survey enabled members of the African Precision Medicine Task Force to develop a Genomic Medicine Framework document, through which we explored different topics related to application of genomic medicine (GM) in Africa. This document has been adopted by the African Academy of Sciences.

It was concluded that implementation of genomic medicine in Africa requires interpretation of genetic findings in a clinical context by considering pathology as an intermediate phenotype linking genes with disease. Aligning lifestyle risk factors and drug response with biochemical parameters and histopathology data relevant to the genes tested, has enormous potential for improving delivery of appropriate healthcare; hence, enrichment of the knowledge base relating to African genetic diversity is essential. In addition, better understanding of the genetic underpinnings of health and disease in Africa will help to elucidate shared disease mechanisms in rest-of-world populations. These considerations resulted in the following key recommendations:

- Build strong relationships with health service stakeholders such as government health departments
- Build governance systems for clinical data, such as informed consent processes for research use and standard operating procedures for research access to data, to ensure the data are used appropriately.
- Build relationships to facilitate ethical and consented sharing of routine health data for GM research and ensure feedback of findings and results to health services.
- Ensure that new clinical data collection is done in collaboration with existing health infrastructures to avoid diluting scarce health resources into parallel data ecosystems, and to ensure that all data are used towards providing better healthcare to the participants.
- Integrate GM approaches and the return of results with routine healthcare provision, at individual, community and population levels as appropriate.
- Use data standardisation with existing, commonly used standards.
- Leverage existing REDCap databases and/or database templates, using federated data storage where needed.
- Ensure most effective research use and meta-analyses from collected data, where consents are in place for data mining, transfer and sharing protocols.

Readiness for implementation of these recommendations in Africa is reflected by pockets of knowledge and evidence generation practices, striving to overcome existing barriers in regulatory data governance and policy. Digital platforms that integrate individual variability in genes, lifestyle and other environmental factors relevant to the biochemistry of each person will enable us to move beyond race and ethnicity as a proxy for the root cause of disease. This can be accomplished by a phased approach, extending from point-of-care genetic testing during a genomic counselling session towards creation of the clinical context for advanced next generation technologies, applied across multiple genes and NCD pathways shown to be particularly relevant in the coronavirus 2019 era. By integrating genetics with pathology toward a one health concept, genomics can be brought into the treatment domain as the ultimate goal of PM.
