# Supplementary material for: A View on Genomic Medicine Activities in Africa: Implications for Policy
Source: Front Genet. 2022 Apr 27;13:769919. doi: 10.3389/fgene.2022.769919 (PMC9091728; doi:10.3389/fgene.2022.769919)
Supplement: Supplementary file 2 [file DataSheet1.PDF]

African Precision Medicine Survey

A task force has been established to develop a framework for Precision Medicine in Africa. We would like to determine what precision medicine activities already exist on the African continent. If you are doing any work in this area please complete the survey.
In completing the survey you agree to have this information used for research or reporting purposes. The information may be added to the Precision Medicine Framework document.

* Required

Start this form over.

Information about you

Medical Scientist

1. **Your name: Maritha**

2. **Your institution: Stellenbosch University**

3. **Current role in your institution (check all that apply)** *
   Check all that apply.

   - [ ] Research scientist
   - [ ] Clinical practitioner
   - [ ] Post-graduate student
   - [ ] Administrator
   - [ ] Laboratory / division head
   - [ ] Other: __________

4. **How familiar are you with the concepts of Precision Medicine?** *
   Mark only one oval.

	1	2	3	4	5	
Not at all	◯	◯	◯	◯	◯	I am an expert

5. **Are you now, or are you planning to use Precision Medicine approaches in your work?** *
Mark only one oval.
- I am already doing this
- I am planning to do it in the future
- I am not planning to do this

Information about your institution

6. **What are the primary activities of your institution? (check all that apply)** *
Check all that apply.
- [ ] Biomedical research
- [ ] Other research
- [ ] Clinical care
- [ ] Government agency
- [ ] Other: ______________________

7. **What is the primary research focus of your institution?** *

____

8. **Does your institution have a Precision Medicine program (clinical or research)** *
Mark only one oval.
- Yes
- No
- Not sure

9. **If your institution has a Precision Medicine program, which diseases or patient populations does it target? Feel free to elaborate.**

____
____
____
____
____

10. **If your institution collects data about patients, which of the following statements apply?**
Check all that apply.

- [ ] We collect demographic information
- [ ] We collect clinical information (patient records)
- [ ] We collect genomic information (genotypes, sequencing data)
- [ ] Our patient data are stored in an electronic medical record system
- [ ] We share patient information with other stakeholders for research purposes
- [ ] We share patient information with other stakeholders for clinical care purposes
- [ ] The ethics of our data collection and sharing activities have been independently reviewed and approved

11. **To what infrastructures do you have access?**
Check all that apply.

- [ ] Biobank for preserving patient samples
- [ ] Standard clinical lab
- [ ] Research lab
- [ ] Genomic analysis facility (sequencing and/or genotyping)
- [ ] Computational facility for data analysis
- [ ] Other: ____________________

Precision Medicine in your country

12. **What is the level of awareness of Precision Medicine in your country?**
Mark only one oval.

- ( ) Most clinicians and researchers are ready to embrace the adoption of Precision Medicine concepts
- ( ) Most clinicians and researchers are aware of the concepts, but not ready to adopt them
- ( ) Awareness of Precision Medicine is low, there is a need for more education
- ( ) Other: ____________________

13. **Is your Government investing in a Precision Medicine program or planning for it?**
Mark only one oval.

- ( ) Yes
- ( ) No
- ( ) Not sure

14. **Are there any institutions in your country that are implementing Precision Medicine approaches?**
Check all that apply.

- [ ] Public hospitals
- [ ] Private hospitals / clinics
- [ ] Individual physicians
- [ ] Research institutions
- [ ] Other: ______________________

15. **What are the barriers to implementing Precision Medicine in your country?**
Check all that apply.

- [ ] The additional costs per patient
- [ ] The lack of information about determinants of disease susceptibility in our population
- [ ] The lack of education of our physicians, researchers and care givers
- [ ] The lack of supporting infrastructures
- [ ] The perception that this is a low priority investment for improving the health of our population
- [ ] The fear that this will benefit only a small proportion of the population
- [ ] Concerns about the ethical, legal, social and institutional framework
- [ ] Other: ______________________

16. **What additional training would be required in your country for a Precision Medicine program?**
Check all that apply.

- [ ] Create a formal degree program in Genomic and/or Personalized Medicine for our physicians
- [ ] Train more Genetic Counselors, or create a training program if not already available
- [ ] Train our nurses and primary care givers in the basic concepts of Precision Medicine
- [ ] Create or expand a BS or MSc degree program in computational biology and medicine
- [ ] Send students and physicians abroad for specialized training
- [ ] Train laboratory technicians in genomic and high-throughput technologies
- [ ] Other: ______________________

Wrapping up

Thank you for your time and your input. We want to give you an opportunity to give us your opinion about the potential to develop Precision Medicine approaches in your country and environment.

17. **Please let us know your thoughts and suggestions. Feel free to elaborate.**

Powered by
Google Forms
